# A US Hospital-Perspective Decision-Analytic Model of Once-Weekly Rezafungin Versus Daily Echinocandin Therapy in Hospitalized Adults With Candidemia or Invasive Candidiasis: Potential Cost Savings From Earlier Discharge

**DOI:** 10.1093/ofid/ofag563

**Published:** 2026-09-09

**Authors:** Thomas P Lodise, Nimish Patel

**Affiliations:** Albany College of Pharmacy and Health Sciences, Albany, New York, USA; Skaggs School of Pharmacy and Pharmaceutical Sciences, University of California San Diego, La Jolla, California, USA

**Keywords:** candidemia, cost analysis, echinocandin, invasive candidiasis, rezafungin

## Abstract

**Background:**

Hospitalized adults with candidemia or invasive candidiasis (C/IC) often remain inpatient to complete daily intravenous echinocandin therapy. Rezafungin, a once-weekly echinocandin, may facilitate earlier discharge in clinically stable patients. We evaluated its potential economic impact from a US hospital perspective.

**Method:**

A decision-analytic probabilistic model compared 2 treatment-discharge strategies in 100 hypothetical hospitalized adults with C/IC who had reached clinical stability and were otherwise dischargeable: continued inpatient daily echinocandin therapy until observed discharge versus administration of a single 400 mg dose of rezafungin with earlier discharge. The rezafungin strategy incorporated earlier discharge distributions from a multicenter US study. Costs included antifungal acquisition, inpatient hospitalization during the final 4 hospital days, and peripherally inserted central catheter (PICC)-related costs (2026 US dollars). Deterministic, threshold, and probabilistic sensitivity analyses (10 000 Monte Carlo simulations) were performed, focusing on excess hospital costs.

**Results:**

In the base case, mean excess cost was lower with rezafungin than standard of care (SOC) (−$412 vs $708 per patient), corresponding to savings of $1120 per patient. Savings were greater when SOC required PICC placement (−$2269) versus no PICC placement (−$851). Rezafungin was associated with higher costs when no earlier discharge occurred (+$3471) or discharge occurred only 1 day earlier (+$1475), but became cost saving with ≥2 days earlier discharge (−$556 at 2 days, −$2903 at 3 days, and −$5313 at ≥4 days).

**Conclusions:**

Once-weekly rezafungin reduced hospital costs versus daily echinocandins when it enabled discharge ≥2 days earlier, with greater savings when PICC placement was avoided.

## Introduction

Candidemia and invasive candidiasis (C/IC) are among the most consequential healthcare-associated fungal infections [1, 2], contributing to substantial morbidity, mortality, prolonged hospitalization, and excess healthcare expenditures [3–8]. Despite advances in diagnostics and supportive care, management of hospitalized patients with C/IC still commonly requires extended courses of intravenous (IV) antifungal therapy [9–11]. Echinocandins remain the preferred initial treatment for many patients because of their broad anti-Candida activity, favorable safety profile, and limited drug–drug interactions [9, 11].

Most currently available echinocandins, including micafungin, caspofungin, and anidulafungin, require daily IV administration [9, 11]. Although this is readily accommodated during the acute phase of infection, the need for continued daily IV therapy may persist after patients are otherwise medically stable, becoming a barrier to hospital discharge and often necessitating peripherally inserted central catheter (PICC) placement for outpatient treatment [12–14]. Recent multicenter US data found that nearly 43% of hospitalized adults receiving an echinocandin near discharge had minimal ongoing inpatient needs and were potentially eligible for discharge 1 to ≥4 days earlier, corresponding to a mean of 2.7 ± 1.2 potentially avoidable hospital days. Approximately 15% also underwent PICC placement at or near hospital discharge to facilitate continued outpatient IV echinocandin therapy [14]. These findings are supported by complementary international and prospective trial data. In the European Confederation of Medical Mycology (ECMM) Candida III multinational cohort of 621 patients with candidemia across 64 centers in 20 countries, hospitalization was prolonged solely to complete IV antifungal therapy in 16.1% of patients, by a median of 16 days (interquartile range [IQR], 8 to 28), with an additional 4.3% requiring outpatient IV antifungal therapy [15]. Similarly, in the multinational ReSTORE phase 3 trial, site investigators prospectively indicated that 16% of patients (30/187) could have been discharged a mean of 5.9 days earlier (median, 5; range, 1 to 14) if once-weekly rezafungin had been available in place of daily infusions [16].

Rezafungin is a next-generation echinocandin with an extended half-life that permits once-weekly IV administration [17–21]. This pharmacokinetic profile may provide an opportunity to reduce reliance on daily inpatient antifungal administration and facilitate earlier discharge in selected clinically stable patients, including transition to outpatient parenteral antifungal therapy (OPAT) or completion of therapy with limited additional outpatient dosing [12, 14, 17, 21]. In hospitals operating near capacity, reductions in potentially avoidable inpatient days may also have broader operational implications, including improved bed availability and patient throughput [22–27].

The economic value of rezafungin in this setting depends on whether higher drug acquisition costs are offset by reductions in hospitalization and vascular access utilization. Where clinically appropriate, step-down to oral fluconazole remains the preferred strategy for completing therapy in patients with susceptible isolates who can tolerate oral treatment [9, 11]. However, oral step-down may not be feasible in patients with fluconazole-nonsusceptible isolates, oral intolerance, contraindications, or clinically significant drug interactions [9, 11, 15]. Thus, the potential economic role of once-weekly rezafungin is most relevant to patients for whom continued IV antifungal therapy remains necessary. To date, there has been limited evaluation of the economic implications of this strategy from a US hospital perspective. We therefore developed a decision-analytic probabilistic model to estimate the potential economic impact of substituting once-weekly rezafungin for daily echinocandin therapy in hospitalized adults with C/IC who had reached clinical stability and were approaching discharge.

## METHODS

### Model Overview and Setting

**Figure 1.**
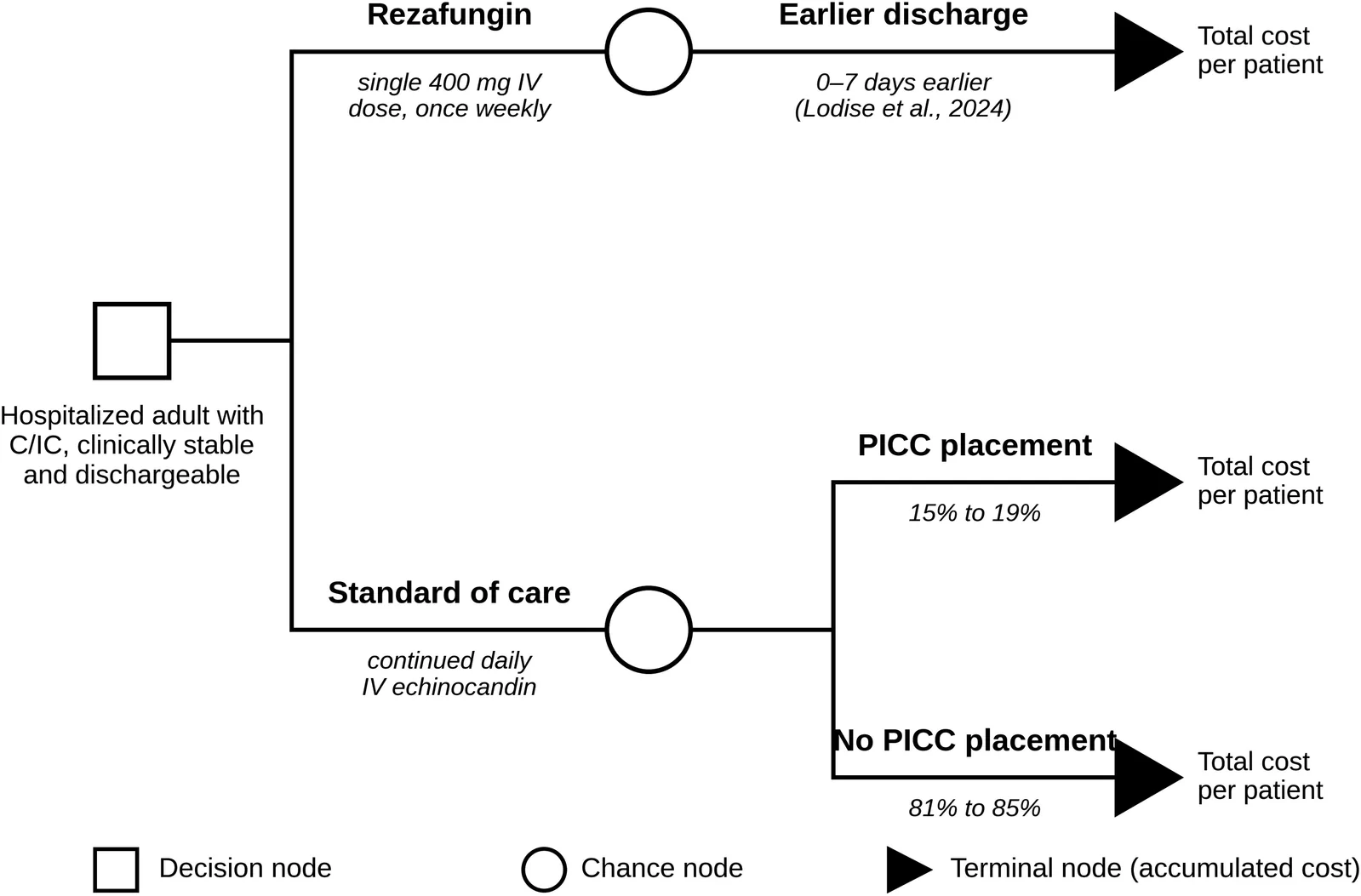
Conceptual structure of the decision analytic model. The decision node (square) represents the choice between once-weekly rezafungin and continued daily echinocandin therapy (standard of care) for a hospitalized adult with candidemia or invasive candidiasis (C/IC) on the first hospital day they were clinically stable and potentially dischargeable. Under the rezafungin strategy, a single 400 mg intravenous dose was administered on the index hospital day; the chance node (circle) represents the probabilistic distribution of days of earlier hospital discharge relative to standard of care (0, 1, 2, 3, 4, 5, 6, or 7 or more days earlier), derived from a multicenter US observational analysis [14]. Because rezafungin does not require daily intravenous administration, no peripherally inserted central catheter (PICC) was assumed to be placed in the rezafungin arm. Under the standard of care strategy, patients continued daily intravenous echinocandin therapy until observed hospital discharge; the chance node represents the probability of PICC placement (15% to 19%) to support ongoing parenteral antifungal administration [14]. Terminal nodes (triangles) represent the accumulated total cost per patient in 2026 US dollars. Full input parameters and unit costs are summarized in Table 1.

We developed a decision-analytic model from the US hospital perspective comparing 2 antifungal treatment-discharge strategies among hospitalized adults with C/IC (Figure 1). The simulated cohort consisted of 100 hypothetical patients who had reached the first hospital day on which they were clinically stable and considered potentially dischargeable, with continued IV echinocandin therapy as the primary remaining barrier to discharge [12–14]. The model time horizon extended from this index day through observed hospital discharge. Model structure and inputs were derived from 3 complementary observational studies that characterized antifungal utilization, discharge timing, healthcare resource use, and inpatient costs near the end of hospitalization [12–14].

Under the standard of care (SOC) strategy, patients continued daily IV echinocandin therapy from the index day until observed hospital discharge. SOC echinocandin use was modeled as a weighted average of micafungin and caspofungin (80% and 20%, respectively), reflecting contemporary US utilization patterns [14]. Under the rezafungin strategy, patients received a single 400 mg IV dose on the index day and were discharged earlier according to discharge distributions derived from the study by Lodise and colleagues [14]. Clinical effectiveness was assumed to be comparable between strategies, supported by the noninferiority of rezafungin to caspofungin in phase 2 and phase 3 trials [19, 20] and was therefore not separately modeled.

### Discharge Timing Inputs

**Figure 2.**
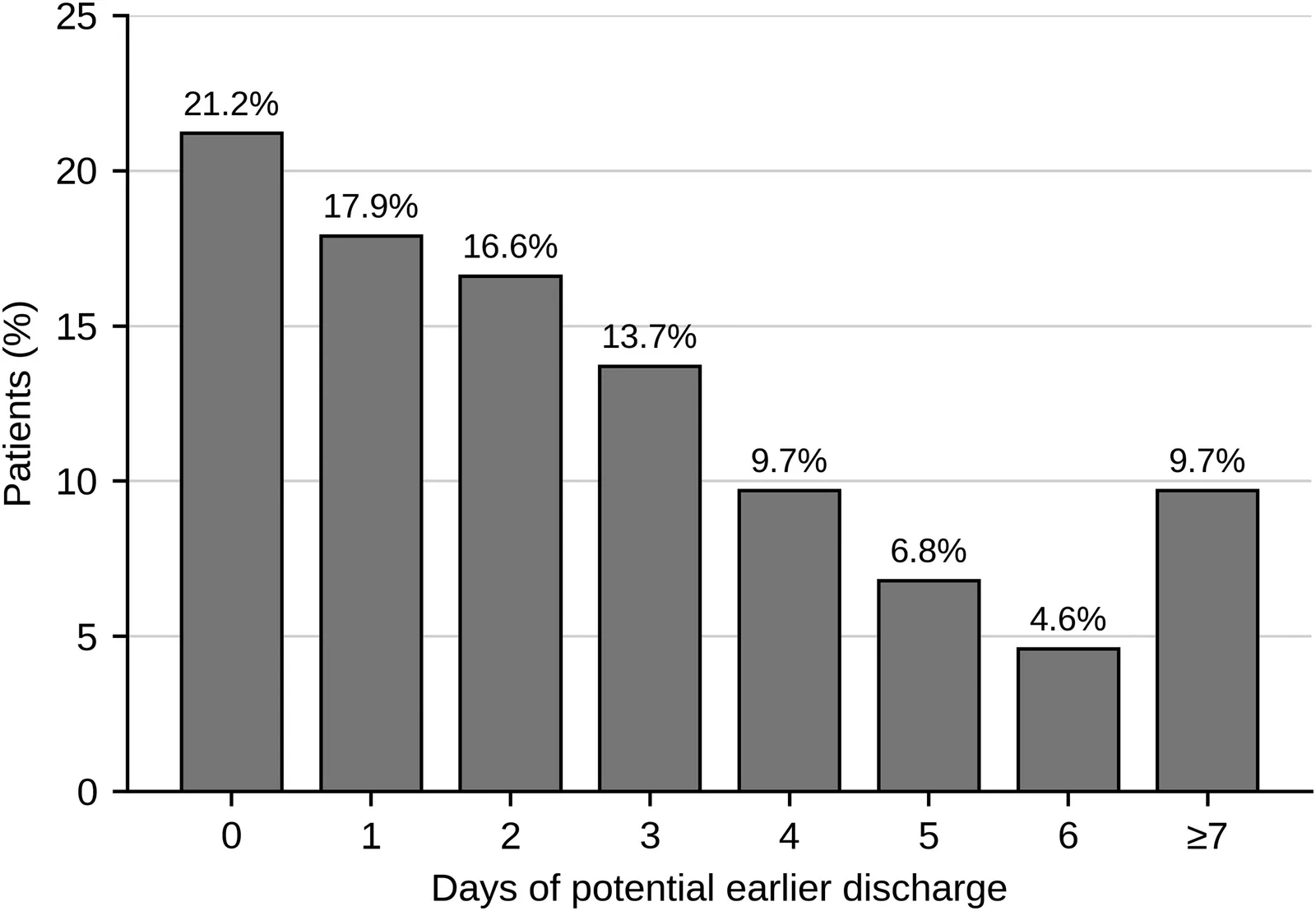
Distribution of potential earlier hospital discharge days enabled by once-weekly rezafungin in the base case. Values represent the modeled proportion of adult patients with candidemia or invasive candidiasis projected to be dischargeable each given number of days earlier than under continued daily echinocandin therapy. Distribution derived from a multicenter US observational analysis [14]. Approximately 79% of patients are projected to be dischargeable ≥1 d earlier than under standard of care.

The distribution of potentially avoidable inpatient days under the rezafungin strategy was informed by a multicenter US observational analysis of 1865 hospitalized adults with C/IC, in which 42.9% of patients receiving an echinocandin near discharge met prespecified criteria for earlier discharge eligibility, with a mean of 2.7 ± 1.2 potentially avoidable hospital days [14]. The base-case distribution for earlier discharge with rezafungin was: 0 days (21.2%), 1 day (17.9%), 2 days (16.6%), 3 days (13.7%), 4 days (9.7%), 5 days (6.8%), 6 days (4.6%), and ≥7 days (9.7%) (Figure 2) [14]. Because hospital discharge often requires coordination of post-acute care and outpatient services, a conservative secondary scenario assumed discharge occurred 1 day after the first potentially dischargeable day to account for residual noninfectious barriers to discharge, including transitions-of-care and home-care logistics described in prior observational studies [12, 28, 29].

### Cost Inputs

Key model inputs are summarized in Table 1. Antifungal acquisition costs (wholesale acquisition cost [30]) were $149.58 per daily dose for micafungin 100 mg, $224.50 per daily dose for caspofungin 50 mg, and $4179.28 per 400 mg dose of rezafungin. The weighted daily SOC acquisition cost was $164.58. Duration of SOC echinocandin therapy from the index day was modeled using a triangular distribution (most likely 2 days; minimum 0; maximum 6 days). Daily inpatient costs during the final 4 hospital days were derived from our prior analysis of the Premier Healthcare Database, a contemporary multicenter US observational cohort of hospitalized adults with C/IC receiving echinocandin therapy [14] and inflated to 2026 US dollars using the medical care component of the Consumer Price Index [31]. Mean daily inpatient costs (SD) were $2092 ($1257) on day −1, $2136 ($1265) on day −2, $2402 ($1469) on day −3, and $2641 ($3104) on day −4. PICC-related costs were applied to a proportion of SOC patients based on observational data indicating that approximately 15% to 19% of patients receiving an echinocandin near discharge underwent PICC placement. Mean PICC insertion and maintenance cost was $1418 (SD $1189; 2026 US dollars), derived from our prior analysis of the Premier Healthcare Database [14] and was modeled using a triangular distribution.

**Table 1.** Key Inputs to the Decision-Analytic Model

Parameter	Value	Source
Cohort size	100 hypothetical hospitalized adults	Model assumption
Index time point	First hospital day patient was clinically stable and potentially dischargeable	Model assumption
**Drug acquisition cost (WAC, 2026 US$)**		
Micafungin 100 mg	$149.58 per daily dose	WAC^a^ [28]
Caspofungin 50 mg	$224.50 per daily dose	WAC^a^ [28]
Standard of care blended cost	$164.58 per daily dose (80% micafungin, 20% caspofungin market share)	Modeled
Rezafungin 400 mg	$4179.28 per dose (administered once on index day)	WAC^a^ [28]
Duration of SOC therapy from index day	Triangular distribution: most likely 2 d; minimum 0; maximum 6 d	Model assumption based on Lodise et al., 2024 [14]
**Daily inpatient cost, mean (SD)**		
Day 1 prior to discharge	$2092 ($1257)	Lodise et al., 2024 [14] (inflated to 2026 US$)
Day 2 prior to discharge	$2136 ($1265)	Lodise et al., 2024 [14]
Day 3 prior to discharge	$2402 ($1469)	Lodise et al., 2024 [14]
Day 4 prior to discharge	$2641 ($3104)	Lodise et al., 2024 [14]
Probability of PICC placement (SOC)	15% to 19% (charge code derived)	Lodise et al., 2024 [14]
PICC insertion plus maintenance cost (2026 US$)	Mean $1418 (SD $1189); triangular distribution	Lodise et al., 2024 [14]
Distribution of earlier discharge (rezafungin arm)	0 d: 21.2%; 1 d: 17.9%; 2 d: 16.6%; 3 d: 13.7%; 4 d: 9.7%; 5 d: 6.8%; 6 d: 4.6%; 7 or more days: 9.7%	Derived from Lodise et al., 2024 [14]

Abbreviations: PICC, peripherally inserted central catheter; SD, standard deviation; SOC, standard of care; US$, US dollars; WAC, wholesale acquisition cost.

^a^WAC values obtained from Red Book [30].

### Analyses

Primary outcomes were mean excess cost per patient and incremental cost differences between strategies, reported overall and stratified by PICC utilization under SOC. Deterministic 1-way sensitivity analyses evaluated the relationship between earlier discharge duration (0, 1, 2, 3, and ≥4 days) and incremental cost. Threshold analyses were additionally performed to estimate the rezafungin acquisition cost at which total expected costs equaled SOC across discharge scenarios. Probabilistic sensitivity analyses using 10 000 Monte Carlo simulations simultaneously varied key model parameters, including antifungal duration, inpatient costs, PICC utilization and costs, and earlier-discharge distributions, across prespecified distributions to assess model robustness. Analyses were conducted in TreeAge Pro Healthcare (Williamstown, MA, USA). Because the model used published aggregate data and did not include individual patient-level information, institutional review board approval was not required.

## RESULTS

### Base-Case Analysis

**Figure 3.**
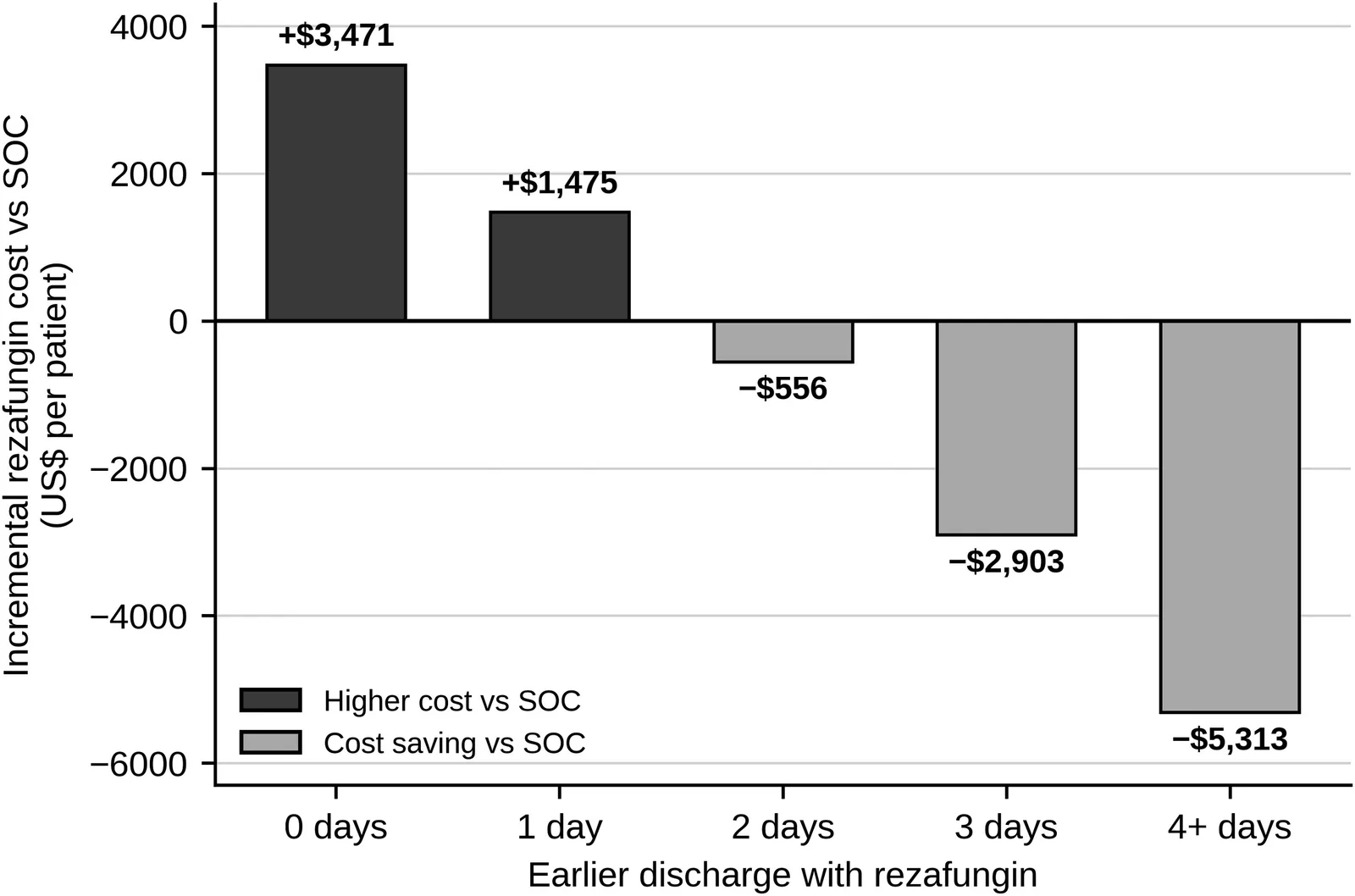
Per-patient incremental cost of rezafungin versus standard daily echinocandin therapy, by days of earlier hospital discharge. Values represent mean incremental cost per patient (US$, 2026). Positive values (red) indicate higher cost with rezafungin; negative values (blue) indicate cost savings. The break-even point is reached at approximately 2 d of earlier discharge.

In the base-case analysis, the modeled mean excess cost per patient was $708 under continued daily echinocandin therapy versus −$412 under once-weekly rezafungin, corresponding to incremental savings of $1120 per patient favoring rezafungin (Table 2). The economic impact of rezafungin was strongly influenced by the magnitude of earlier discharge. When no earlier discharge occurred, rezafungin was associated with higher costs than SOC (+$3471 per patient). With only 1-day earlier discharge, rezafungin also remained more costly (+$1475 per patient). However, rezafungin became cost saving when discharge occurred ≥2 days earlier (−$556 per patient at 2 days), with progressively greater savings at 3 days (−$2903) and ≥4 days (−$5313) earlier discharge (Figure 3). Applying the base-case earlier-discharge distribution, 60.9% of patients were projected to achieve the ≥2-day earlier-discharge threshold at which rezafungin became cost saving, whereas 21.2% derived no discharge benefit and 17.9% were discharged only 1 day earlier and therefore remained cost additive.

**Table 2.** Base-Case Mean Weighted Costs Per Patient and Incremental Cost Differences (US$, 2026)

Strategy	Mean Weighted Cost (US$)	Rezafungin vs SOC Overall	Rezafungin vs SOC With PICC	Rezafungin vs SOC Without PICC
**SOC, overall**	**$708**	—	—	—
SOC, with PICC	$1857	—	—	—
SOC, without PICC	$439	—	—	—
**Rezafungin (base case)**	**−$412**	**−$1120**	**−$2269**	**−$851**
DC 0 d earlier	$4179	+$3471	+$2322	+$3740
DC 1 d earlier	$2183	+$1475	+$326	+$1744
DC 2 d earlier	$152	−$556	−$1705	−$287
DC 3 d earlier	−$2195	−$2903	−$4052	−$2634
DC 4+ days earlier	−$4605	−$5313	−$6462	−$5044

Abbreviations: DC, discharge; PICC, peripherally inserted central catheter; SOC, standard of care.

Highlighted row indicates the overall base-case rezafungin estimate. Negative values indicate cost savings versus standard of care.

Savings associated with rezafungin were greater when SOC required PICC placement (−$2269 per patient) compared with scenarios without PICC placement (−$851 per patient), reflecting avoided vascular access procedures and maintenance costs. The incremental economic advantage of rezafungin was preserved across all earlier-discharge scenarios of ≥2 days (Table 2).

### Conservative Discharge Scenario

In the prespecified conservative scenario, patients in the rezafungin strategy were assumed to be discharged 1 day after first meeting discharge eligibility criteria to account for residual noninfectious barriers to discharge. Under this assumption, rezafungin remained economically favorable, with mean excess costs of −$61 per patient versus $708 per patient under SOC, corresponding to incremental savings of $769 per patient. Similar to the base-case analysis, savings were greater when PICC placement was avoided (−$1918 per patient versus SOC with PICC placement and −$500 per patient versus SOC without PICC placement) and increased with longer durations of earlier discharge.

### Threshold Analyses

**Figure 4.**
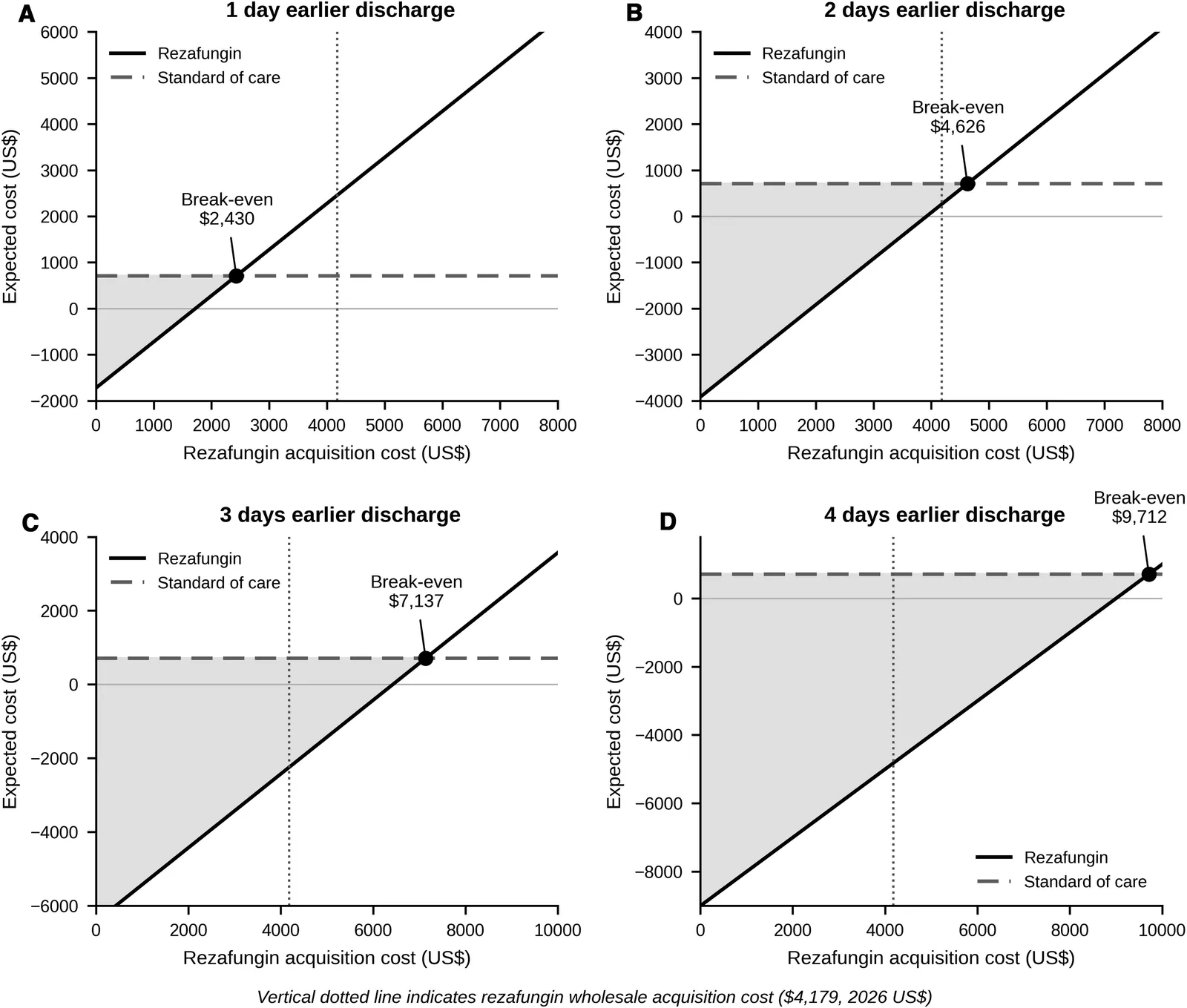
Threshold analyses of rezafungin acquisition cost versus standard of care, by days of earlier hospital discharge. Each panel shows the expected per-patient cost (US$, 2026) of rezafungin (blue line) and continued daily echinocandin standard of care (SOC, orange line) as a function of rezafungin acquisition cost, assuming a fixed level of earlier discharge: (*A*) 1 d, (*B*) 2 d, (*C*) 3 d, (*D*) 4 d. Filled markers indicate the break-even acquisition cost at which expected total cost with rezafungin equals SOC: $2430 (*A*), $4626 (*B*), $7137 (*C*), and $9712 (*D*). The vertical dotted line indicates the modeled rezafungin wholesale acquisition cost ($4179, 2026 US). Rezafungin is below break-even, and therefore cost-saving, whenever earlier discharge is 2 or more days. Shaded regions indicate cost-saving conditions for rezafungin.

Threshold analyses identified the rezafungin acquisition cost at which total expected costs were equivalent to SOC across earlier-discharge scenarios (Figure 4). Estimated break-even acquisition cost thresholds for rezafungin were approximately $2430 with 1-day earlier discharge, $4626 with 2 days, $7137 with 3 days, and $9712 with 4 days earlier discharge. At the modeled 2026 wholesale acquisition cost of $4179.28, rezafungin remained below the break-even threshold when earlier discharge was at least 2 days.

### Probabilistic Sensitivity Analyses

In the base-case probabilistic sensitivity analysis (10 000 Monte Carlo simulations), mean modeled excess cost per patient was −$419 (SD $440; median −$406; IQR, −$718 to −$109) for rezafungin versus $707 (SD $303; median $698; IQR, $495 to $912) for SOC (Table 3). In the conservative-scenario probabilistic sensitivity analysis, mean excess cost per patient was −$65 (SD $409) for rezafungin versus $703 (SD $302) for SOC. Across both analyses, the direction and magnitude of incremental cost differences consistently favored rezafungin despite simultaneous variation in key model parameters.

**Table 3.** Probabilistic Sensitivity Analysis (10 000 Monte Carlo Simulations)

Statistic	Base Case: Rezafungin	Base Case: SOC	Scenario: Rezafungin	Differences, Rezafungin vs SOC
Simulations, n	10 000	10 000	10 000	…
**Mean cost (US$)**	−$419	$707	−$65	−1126
Standard deviation	$440	$303	$409	535
Minimum	−$1988	−$298	−$1443	−3061
25th percentile	−$718	$495	−$345	−1485
Median	−$406	$698	−$53	−1114
75th percentile	−$109	$912	$222	−751
Maximum	$1081	$1893	$1257	922

Abbreviation: SOC, standard of care.

Negative values indicate net cost savings per patient under the modeled strategy.

## DISCUSSION

In this US hospital-perspective decision-analytic model, once-weekly rezafungin was associated with lower expected costs than continued daily echinocandin therapy among clinically stable hospitalized adults with C/IC, when its use enabled earlier discharge by at least 2 days, with additional savings when PICC placement was avoided. Estimated mean savings were $1120 per patient in the base-case analysis and $769 per patient in a conservative scenario incorporating residual noninfectious barriers to discharge. Reductions in inpatient length of stay were the primary driver of economic benefit, while avoidance of PICC placement contributed additional value.

These findings may have practical implications for hospital formulary committees and discharge-management programs considering once-weekly antifungal therapy [22–27, 32]. The model suggests that the higher acquisition cost of rezafungin may be offset by avoided hospitalization costs when antifungal selection meaningfully alters discharge timing. In contrast, when hospitalization would continue regardless of antifungal administration requirements, the economic advantage of rezafungin was substantially attenuated. These findings support a targeted implementation approach focused on clinically stable patients whose continued hospitalization is primarily related to the need for ongoing daily IV antifungal therapy and/or anticipated PICC placement [32]. Threshold analyses further demonstrated that the rezafungin acquisition cost associated with cost neutrality increased progressively with each additional avoided inpatient day.

Our findings are also consistent with broader healthcare-system priorities emphasizing reduced avoidable hospitalization and improved inpatient throughput [33, 34]. In contemporary hospital systems operating near capacity, reductions in inpatient length of stay may provide operational benefits extending beyond direct hospitalization costs, including improved bed availability and patient flow [24–27, 33, 34]. These downstream operational effects, along with reduced exposure to nosocomial complications associated with prolonged hospitalization, were not explicitly incorporated into the current economic model [3].

Several limitations should be considered when interpreting these findings. This was a decision-analytic model informed by limited observational data [12–14], and real-world implementation may differ from modeled assumptions. Institutions seeking to evaluate the economic impact of rezafungin should therefore consider adapting model inputs to local practice patterns and costs. Clinical effectiveness was assumed to be comparable between strategies based on the noninferiority of rezafungin to caspofungin in the ReSTORE trial and was not separately modeled [19]. Antifungal acquisition costs were based on wholesale acquisition cost [30] and may differ from actual institutional acquisition costs because of contracting arrangements, rebates, or 340B participation.

A key uncertainty is the extent to which once-weekly rezafungin translates into earlier discharge in routine clinical practice. Direct evidence from ReSTORE and STRIVE is limited because both trials used double-blind, double-dummy designs in which patients in both treatment groups continued to receive daily infusions [19, 20]. As a result, these trials were not designed to directly evaluate whether the once-weekly dosing schedule itself facilitated earlier hospital discharge, because patients in both treatment arms remained hospitalized to receive daily study infusions [16]. Consequently, any observed differences in length of stay should be interpreted cautiously. A pooled exploratory analysis of ReSTORE and STRIVE nevertheless found numerically shorter hospital stays with rezafungin (mean total hospital stay, 25.2 vs 28.3 days; mean intensive care unit [ICU] stay, 16.1 vs 21.6 days; adjusted ICU difference, 4.1 days [relative difference, 24%; 95% CI, −11% to 72%]), although the estimates were imprecise [16]. More directly relevant to the present model, prospectively collected investigator assessments in ReSTORE indicated that 16% of patients could have been discharged a mean of 5.9 days earlier if once-weekly rezafungin had been available without the requirement for daily study infusions [16]. This estimate is consistent with the independent ECMM Candida III finding that hospitalization was prolonged solely to complete IV antifungal therapy in 16.1% of patients [15].

Emerging post-approval experience is also consistent with use of rezafungin to facilitate outpatient antifungal therapy and hospital discharge. In a multinational registry, enabling OPAT was the primary reason for selecting rezafungin in 86% of patients [35]. In an early access program involving 6 patients with chronic, difficult-to-treat C/IC in Italy and Germany, rezafungin was administered once weekly for 5 to 39 weeks during the program, allowing treatment to continue in the outpatient setting and facilitating hospital discharge in patients who otherwise required ongoing IV antifungal therapy [36]. In a single-center Spanish cohort, facilitating outpatient antifungal therapy was a stated reason for initiation in 30.8% of patients [37]. These reports are small, uncontrolled, and heterogeneous and should therefore be considered supportive rather than confirmatory. Real-world comparative data quantifying actual reductions in discharge timing and hospital costs following rezafungin implementation remain unavailable, underscoring the need for prospective validation.

The analysis focused on the inpatient discharge phase and did not comprehensively capture outpatient treatment and follow-up costs after discharge. This omission may bias the analysis conservatively against rezafungin, as many hospitalized adults with C/IC receiving standard echinocandin therapy continue daily IV treatment after discharge [12–14], whereas once-weekly rezafungin may complete therapy with a single inpatient dose or substantially fewer outpatient infusions. The analysis also did not account for potential care-pathway efficiencies associated with outpatient continuation or initiation of once-weekly rezafungin therapy, which may further favor rezafungin relative to daily echinocandin regimens. Patients receiving outpatient echinocandin therapy accrue additional costs not included in the model, including antifungal acquisition, OPAT nursing and pharmacy services, PICC placement and maintenance, and catheter- or infusion-related complications that may result in emergency department visits or readmissions [32, 38–40]. Because these costs scale with infusion frequency (approximately 7 infusions/week for daily echinocandins vs 1/week for rezafungin), their exclusion may underestimate the overall economic benefit of rezafungin across the continuum of care.

Relatedly, the model did not assess patient-reported outcomes, health-related quality of life, or treatment satisfaction. These domains are particularly relevant because the 2 strategies appear to differ less in clinical efficacy, as suggested by the ReSTORE noninferiority results [19], than in treatment burden. Daily IV echinocandin therapy, whether completed during hospitalization or through OPAT, requires repeated infusions and often placement of an indwelling vascular catheter, which can limit mobility and daily activities while increasing the need for caregiver involvement in line care, medication administration, and infusion coordination [32, 38–40]. In contrast, once-weekly rezafungin reduces infusion frequency and may avoid vascular access placement entirely, potentially decreasing treatment burden and improving the patient experience. Because these effects were neither measured nor incorporated into the economic model and would most plausibly favor rezafungin, these unmeasured effects may represent additional value not captured in the economic model. Prospective studies incorporating validated quality-of-life and treatment satisfaction measures are warranted to better characterize this dimension of value.

Finally, generalizability may vary across institutions because of differences in discharge processes, payer structures, and hospital bed-capacity dynamics. Although the conservative scenario incorporated residual noninfectious discharge barriers, additional real-world barriers such as insurance authorization or post-acute placement delays may further attenuate realized savings. Despite these limitations, findings were robust across sensitivity analyses. The economic value of rezafungin is likely greatest in clinically stable C/IC patients whose continued hospitalization is driven by daily IV echinocandin administration, particularly when PICC placement would otherwise be required [14]. Real-world validation of these projections will require prospective evaluation of discharge timing and total episode costs among clinically stable patients transitioned to once-weekly therapy, with concurrent controls receiving daily echinocandins and explicit capture of noninfectious discharge barriers. Until such data are available, the present model is best interpreted as an institutional decision aid rather than a general claim regarding the value of rezafungin across an unselected C/IC population. Institutions can adapt model inputs to local practice patterns and costs and use the threshold analyses to identify their own break-even point.

In conclusion, once-weekly rezafungin was associated with a favorable hospital economic profile compared with daily echinocandin therapy when its use enabled earlier discharge, with cost savings emerging when discharge occurred at least 2 days earlier and greater savings when PICC placement was avoided. The projected savings were driven primarily by reductions in inpatient length of stay, with additional benefit from avoidance of PICC placement. These findings support targeted use in patients whose continued hospitalization is primarily driven by the need for ongoing daily IV antifungal therapy. In current practice, oral step-down to fluconazole remains the preferred approach for patients with azole-susceptible isolates who can tolerate oral therapy. Accordingly, the economic case for once-weekly rezafungin described here is most relevant to patients for whom oral step-down is not feasible because of azole-nonsusceptible isolates, oral intolerance, contraindications, or clinically significant drug interactions.
